# A CNS-Directed, AAV9 Gene Therapy Restores Expression and Biochemical Function of Guanidinoacetate Methyltransferase in Models of GAMT Deficiency

**DOI:** 10.3390/ijms27021035

**Published:** 2026-01-20

**Authors:** Robyn Binsfeld, Troy Webster, Ilona Tkachyova, Michael Tropak, Melissa Mitchell, Tesla Peretti, Andreas Schulze, Jagdeep S. Walia

**Affiliations:** 1 Centre for Neuroscience Studies, Queen’s University, Kingston, ON K7L 3N6, Canada; 15rb31@queensu.ca (R.B.);; 2 The Hospital for Sick Children, Toronto, ON M5G 1E8, Canadaandreas.schulze@sickkids.ca (A.S.); 3 Peter Gilgan Center for Research and Learning, Toronto, ON M5G 0A4, Canada; 4 Department of Pediatrics, Queen’s University, Kingston, ON K7L 3N6, Canada; 5 Departments of Pediatrics and Biochemistry, University of Toronto, Toronto, ON M5S 1A1, Canada

**Keywords:** scAAV9.hGAMT therapy, gene therapy, creatine deficiency, AAV9, intrathecal, GAMT deficiency

## Abstract

Guanidinoacetate methyltransferase (GAMT) is an essential enzyme in the biosynthesis of creatine, an important molecule in energy recycling. GAMT loss of function leads to GAMT deficiency (GAMT-D), an autosomal recessive disorder resulting in low creatine levels and the accumulation of a toxic intermediate, guanidinoacetate (GAA). GAMT-D patients present with intellectual disability and epilepsy, emphasizing the detrimental consequences of disturbed creatine metabolisms in the central nervous system (CNS). Current treatments are not curative and may not restore creatine metabolism in the brain. Here, we present a proof-of concept study testing the first CNS-directed, Adeno-associated virus serotype 9 (AAV9)-based gene therapy for the treatment of GAMT-D. the delivery of *GAMT* construct to cellular models of GAMT-D effectively restored protein and mRNA expression of GAMT while increasing intracellular creatine content and decreasing GAA accumulation. In murine models of GAMT-D, treatment with *scAAV9.hGAMT,* delivered intrathecally, resulted in increased creatine content as well as significant decreases in GAA accumulation in the CNS and peripheral organs. Overall, we found that *scAAV9.hGAMT* represents a promising gene therapy for treating GAMT-D, warranting further investigation in animal models to determine an appropriate therapeutic window for both efficacy and safety that allows for translation into human patients in the future.

## 1. Introduction

**Figure 1:**
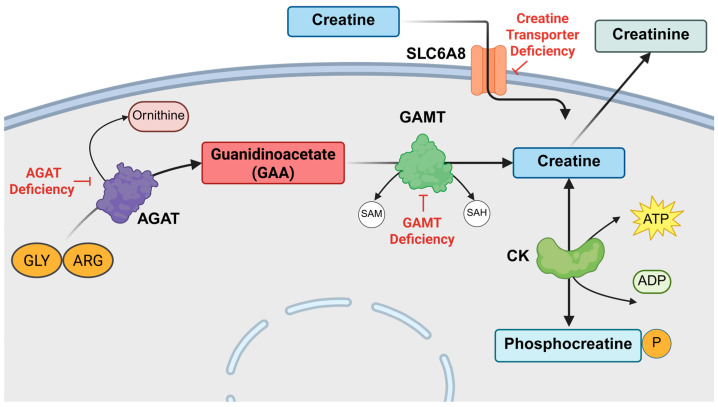
Creatine biosynthesis and creatine deficiency syndromes. Arginine and glycine are enzymatically converted to ornithine and guanidinoacetate by L-arginine:glycine amidinotransferase (AGAT). AGAT deficiency (AGAT-D) results from loss of function of the AGAT enzyme, leading to low levels of GAA and Cr. GAA is transformed into creatine through the addition of a methyl group by guanidinoacetate methyltransferase (GAMT) via the conversion of S-adenosyl-l-methionine to S-adenosyl-l-homocysteine. GAMT deficiency (GAMT-D) results from loss of function of the GAMT enzyme, leading to accumulation of the GAA substrate and low Cr production. Creatine can then be imported into other cells through the creatine transporter, SLC6A8. Creatine transporter deficiency occurs when there is a loss of function of SLC6A8 and leads to reduced creatine uptake in muscle and the brain. Creatine will enter the phosphocreatine system to participate in energy recycling via creatine kinase (CK). Creatine and phosphocreatine are non-enzymatically converted to creatinine for excretion in urine.

Creatine’s (Cr) primary function is in energy metabolism through the recycling of adenosine triphosphate (ATP) via the phosphocreatine–creatine system [1,2,3,4,5]. The reversible reaction of Cr to its phosphorylated form enables the phosphorylation of adenoside diphosphate (ADP) to ATP for the energetic processes of the cell [5]. Cr has been widely known for its role in muscle function, reported in athletes; however, it has been shown to play an important role in brain function and development. Cr may be obtained through diet or synthesized endogenously via two enzymatic steps and a transport system, as detailed in Figure 1. L-arginine:glycine amidinotransferase (AGAT), encoded by the gene *GATM*, converts arginine and glycine to ornithine and guanidinoacetate (GAA). The guanidinoacetate methyltransferase (GAMT) enzyme, encoded by the *GAMT* gene located at the 19p13.3 locus, catalyzes the methylation of GAA in the presence of S-adenosyl-l-methionine (SAM) [6,7]. GAMT has been confirmed to have a core alpha/beta open-sandwich structure common to methyltransferases, with its active site uniquely obscured by a flexible N-terminal section at residues 1–42 [6]. When the N-terminal section moves to open the active site, it allows GAA and SAM to enter and bind through extensive hydrogen bonding and hydrophobic interactions [6]. Once the active site closes to exclude water, Asp134 abstracts a proton from the N_ε_ of GAA to create a strong nucleophile, which then attacks the methyl group of SAM to produce creatine and S-adenosylhomocysteine (SAH) [6].

In the periphery, Cr synthesis takes place primarily in the liver with the formation of GAA in the kidneys. Cr enters the systemic circulation from the liver and is imported into other tissues by solute carrier family 6 member 8 (SLC6A8, CTR). It has been shown that the brain compartment contains all components required for Cr synthesis and transport. AGAT and GAMT are co-expressed in fewer than 20% of cells in the brain; therefore, GAA must exit neuronal cells and be imported through SLC6A8 or γ-aminobutyric acid (GABA) transporters to cells that express GAMT [8,9,10,11,12,13,14]. SLC6A8 is not expressed in astrocytes, resulting in low permeability of Cr through the blood–brain barrier (BBB) [12,15]. This suggests that the brain relies heavily on endogenous synthesis for its Cr supply, which explains the need to supplement to supraphysiological doses of Cr in GAMT or AGAT deficient patients to replenish brain Cr.

Three disorders of Cr metabolism have been discovered due to loss of function of AGAT, GAMT, or SLC6A8. All of the cerebral Cr deficiency syndromes (CCDS) have low levels of brain Cr and several neurological manifestations [2,3]. GAMT deficiency (GAMT-D, CCDS2, OMIM 612736) is an autosomal recessive genetic disorder leading to low levels of Cr and the accumulation of GAA in the brain and periphery [16,17,18]. Symptoms include developmental delay, intellectual disability, epilepsy, and behavioural disorders. GAMT-D is the most severe form of CCDS, often attributed to the additional accumulation of GAA and its potential neurotoxic and epileptogenic effects [15,16,19,20,21]. There have been approximately 130 people diagnosed with GAMT-D to date. GAMT-D is considered an ultra-rare disease, with an incidence estimated to be between 1:2,640,000 and 1:250,000 [7,22,23,24]. Recent newborn screening programmes have shed light onto the incidence of GAMT, confirming this window, with specific studies showing a 1:405,665 rate in New York and Utah [23]. The only approved treatment for GAMT-D consists of significant oral Cr monohydrate (250–1000 mg/kg) and ornithine supplementation in combination with arginine restriction to control the accumulation of GAA [25,26]. This is often successful at limiting some symptoms, especially seizures; however, neurodevelopmental symptoms often remain unless treatment is initiated immediately after birth [26,27,28]. The treatment is also difficult to maintain, and compliance is often an issue, especially in older patients [29]. Additional treatment approaches include symptom management in the form of behavioural and speech therapy. Given the importance of early intervention, GAMT-D has been recently added to several newborn screening panels in the US and Canada [30,31].

Recently, a rh10-AAV-based gene therapy using a liver-specific promoter to treat GAMT deficiency in mice was investigated that effectively restored Cr and reduced GAA levels in several tissues [32]. This is a promising step for the treatment of GAMT-D; however, the direct intrathecal delivery of an AAV9 vector and the use of a ubiquitous promoter may present a more curable approach to the disease, given the neurological presentation seen in humans. Due to the natural tropism of AAV9, its use has the potential to treat the disease both systemically and lead to direct expression of the transgene in the cells of the CNS. The intravenous delivery of AAV-mediated therapies also requires higher doses to achieve therapeutic efficacy, which may elicit strong immune responses that could ultimately render the vector ineffective and are far more costly in a clinical setting [33,34,35,36]. Localized delivery directly to the CNS via an intrathecal route has a greater ability to avoid neutralizing antibodies and requires smaller doses, with the potential to achieve similar or increased therapeutic benefit [37]. Here, we designed and investigated the first intrathecal gene therapy approach for treating GAMT-D using a self-complementary AAV9 vector with the synthetic ubiquitous JeT promoter. In mice, the localized dose, delivered via lumbar puncture, was over 2000-fold lower than a typical intravenous dose. We show that the designed *scAAV9.hGAMT* can effectively restore the protein expression of GAMT while increasing intracellular Cr content and reducing GAA accumulation in both cellular and murine models of GAMT-D. This effect was observed in both the periphery and the CNS of treated animals, indicating that the vector can effectively address the overall pathology of GAMT-D following intrathecal delivery.

## 2. Results

### 2.1. An In Vitro ‘Proof-of-Concept’ Study Shows Restored GAMT Expression and Intracellular Cr Content in a Treated Cellular Model of GAMT-D

To confirm that the designed construct could successfully restore GAMT expression when delivered to cells, in vitro experiments were first performed with plasmid DNA containing the human *GAMT* (phGAMT) construct packaged within inverted terminal repeats (ITRs). A cellular model of GAMT-D was generated in a HAP1 cell line (generously provided by Andreas Schulze, University of Toronto, Canada). For all the below experiments, wildtype (WT) HAP1 cells were used as positive controls while knockout (KO) cells treated with a plasmid containing *GFP* only were used as negative controls. For these experiments, cells were grown in media supplemented with dialyzed FBS to limit the presence of small molecules such as Cr that could impact the results, particularly analyses of Cr and GAA content.

#### 2.1.1. GAMT Protein and Gene Expression Are Restored Following Transfections of the Cellular Model of GAMT-D with Plasmid Carrying the Designed GAMT Construct

**Figure 2:**
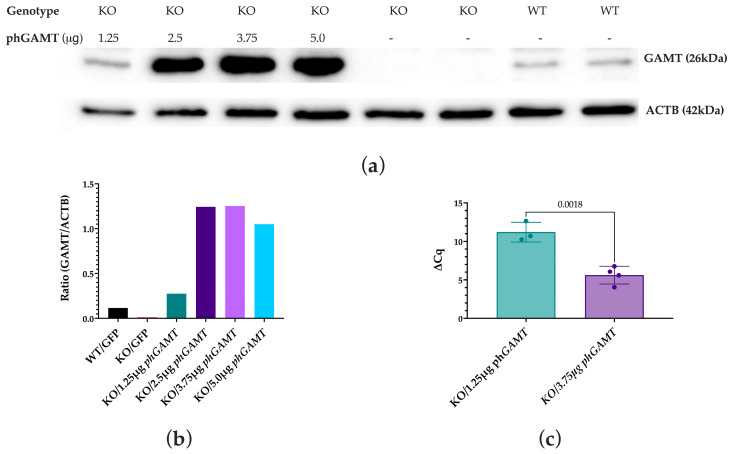
Protein and mRNA expression of GAMT following transfection with phGAMT. (**a**). Following transfections with ph*GAMT*, protein lysates were collected and ran on a Western blot using approximately 30 μg of protein per well. In lane 1, a low dose of 1.25 μg of plasmid DNA was used. In lane 2, a dose of 2.50 μg was used. In lane 3, a dose of 3.75 μg was used, and in lane 4, a dose of 5.00 μg was used. Lanes 5–6 represent negative controls of KO cells transfected with a plasmid only expressing *GFP*. Lanes 7–8 consist of a positive control of untransfected WT HAP1 cells. Expression was restored to that greater than WT cells and started showing saturation at approximately 2.5 μg. (**b**). A quantification of the protein expression in (**a**) shown as a ratio of GAMT:ACTB expression. Data is represented by the mean or a single data point where applicable. (**c**) Knockout cells were transfected with either a high dose (3.75 µg) or low dose (1.25 µg) of phGAMT or a GFP plasmid control. RNA was isolated and then reverse transcribed into cDNA for use in qPCR. The low dose was used as a normalization point for all samples as the untreated sample does not have a detectable level of the codon-optimized plasmid. The delta Cq value of the high dose was significantly lower than the low dose, indicating a higher level of GAMT mRNA in the samples. An approximate 1.5-fold difference was observed in gene expression between the two treatment groups (*p* = 0.0018). Data is represented as the mean (SD) and an unpaired *t*-test was run to compare the two treatment groups.

**Figure 3:**
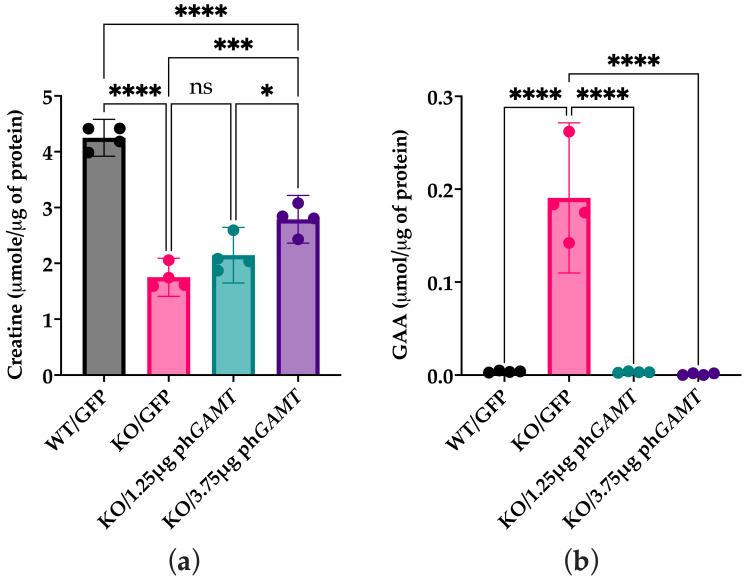
Intracellular Cr and GAA content following low-dose and high-dose transfection of KO GAMT cells with phGAMT. (**a**) There was no significant difference observed in intracellular Cr concentration between the low-dose treated and untreated KO cells; however, the high dose (3.75 μg) of ph*GAMT* resulted in a significant increase in Cr compared to GFP-treated KO cells. The high dose did not restore Cr to the wildtype levels, likely due to lack of 100% transfection rate. (*n* = 4, * *p* < 0.02, *** *p* < 0.0002, **** *p* < 0.0001, ns = not significant). (**b**). The treatment of GAMT knockout cells with both the low (1.25 μg) and high (3.75 μg) dose significantly reduced GAA accumulation, comparable to that observed in WT HAP1 cells (*n* = 4, *p* < 0.0001). Data is represented by the mean (SD) and a one-way ANOVA was used to compare groups.

To confirm protein expression could be restored following the transfection of *GAMT* KO cells, a Western blot was performed. *GAMT* KO cells were transfected with the *hGAMT* plasmid at a range of dosages from 1.25 μg to 5.0 μg of plasmid DNA. This range of dosages was determined based on the transfection reagent protocol and to test for the optimal plasmid delivery. All doses resulted in detectable GAMT protein expression at 1.25, 2.5, 3.75, and 5.0 μg (Figure 2a,b). Saturation of the blot was observed between 2.5 and 3.75 μg; thus, going forward, the 3.75 μg was chosen as the high dose for the further experiments detailed in Figure 2c and Figure 3. RNA was isolated from transfected cells to assess gene expression by RT-qPCR. The reference gene *RNA18S* was used to quantify gene expression using the ΔCq method (Figure 2c). The gene expression of *hGAMT* was detectable in both doses following treatment of knockout cells with ph*GAMT*, with an approximate 1.5-fold increase in expression between the low- and high-dose treatment groups (Figure 2c). It was evident that the transfection of *GAMT* KO cells with the designed *GAMT* plasmids successfully restored protein and mRNA expression, with greater expression at the higher doses.

#### 2.1.2. Intracellular Cr Content Is Increased While GAA Content Is Decreased in Treated Cellular Models of GAMT-D

Cr and GAA were measured via liquid chromatography tandem mass spectrometry (LC-MS/MS) in KO cells transfected with the low and high doses of ph*GAMT*. A significant reduction in intracellular GAA was observed at all doses (Figure 3b). However, intracellular Cr was only increased significantly in the 3.75 µg dose group (Figure 3a).

### 2.2. An In Vivo ‘Proof-of-Concept’ Study in Murine Models of GAMT-D Shows That Intrathecal Treatment with scAAV9.hGAMT Effectively Restores GAMT Expression and Intracellular Cr and GAA Levels Compared to Untreated Controls

*Gamt* KO mice were treated with an intrathecal lumbar puncture administration of scAAV9.*hGAMT* at a dose of 1.1 × 10^11^ vector genomes (vg) per mouse at 6 weeks of age (*n* = 7). A vehicle solution was delivered intrathecally to heterozygous (*n* = 6) and KO (*n* = 5) mice as positive and negative controls, respectively. Mice were immunosuppressed from five weeks of age until their endpoint at 13 weeks of age or 7 weeks post injection, at which point tissues were collected for analysis of guanidino compounds, vector biodistribution and GAMT protein expression.

#### 2.2.1. Cr Content Is Significantly Increased While Gaa Is Significantly Decreased in Several Tissues Including the CNS in Treated Mice Compared to Untreated Controls

**Figure 4:**
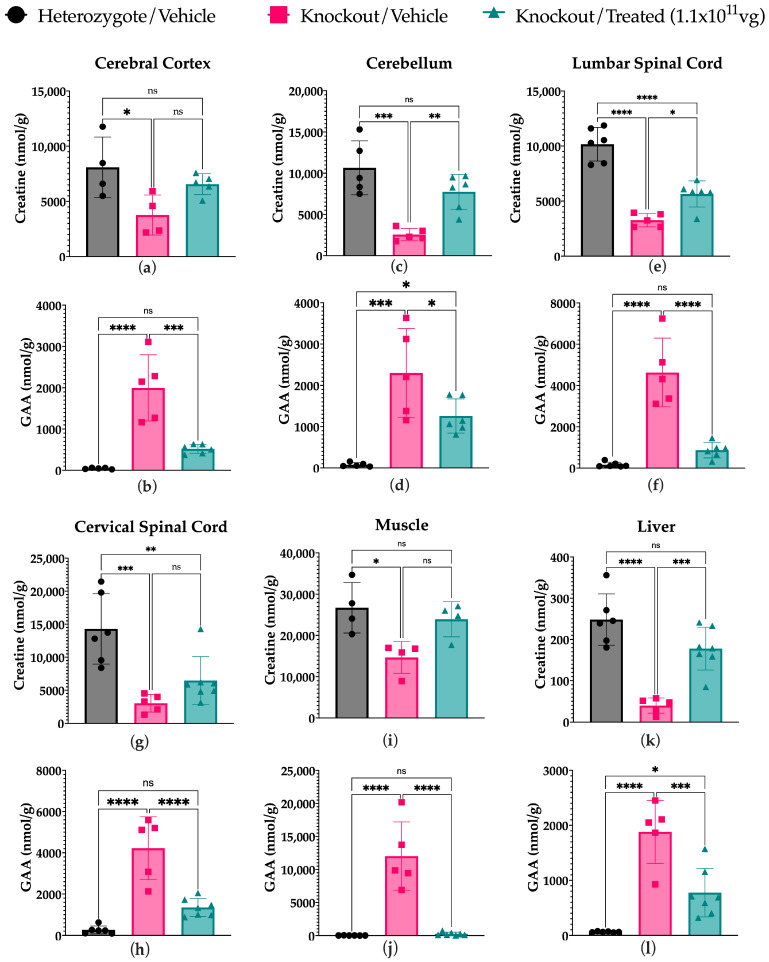
Intracellular Cr and GAA content in tissue samples of murine models of GAMT-D treated with scAAV9.hGAMT and vehicle controls determined by LC-MS/MS. Mice were treated with either 1.1 × 10^11^ vg/mouse or a vehicle solution by intrathecal lumbar puncture. Tissues were collected at endpoint and proteins were isolated for analysis by LC-MS/MS for guanidino compounds. An observable trend of increased Cr content in treated mice was observed compared to vehicle-treated knockouts. This increase was found to be significant in the cerebellar section of the brain (**c**) and lumbar section of the spinal cord (**e**), as well as the liver (**k**); however, in the muscle (**i**), cervical spine (**g**), and cerebrum (**a**) it was not significantly increased. There were also no significant differences between the treated mice and the heterozygote controls in these tissues, except for the cervical spinal cord. A significant decrease in GAA accumulation was observed in treated mice compared to vehicle-treated knockouts in all tissues tested (**b**,**d**,**f**,**h**,**j**,**l**). In most cases, this was normalized to heterozygote levels, with the exception of the cerebellum (**d**) and the liver (l) (*n* = 4–7, * *p* < 0.05, ** *p* < 0.007, *** *p* < 0.0007, **** *p* < 0.0001, ns = not significant). Data is represented as the mean (SD) and a one-way ANOVA was used to compare groups.

Several tissues were collected, including three sections of the brain and two sections of the spinal cord, in addition to muscle and liver samples. Serum was collected at endpoints as well as monthly, starting at a baseline of 5 weeks prior to injections. We observed a trend showing increased intracellular Cr in all tissue types; however, this was only significant (*p* < 0.05) in the cerebellum, lumbar spinal cord, and liver (Figure 4c,e,k, Table S1). Cr levels were not significantly higher than vehicle-treated *Gamt* KO in the other tissues tested, including the cerebral cortex, cervical spinal cord, and muscle (Figure 4a,g,i). However, there were also no significant differences observed between the treated *Gamt* KO mice and the heterozygotes in the muscle and cerebral cortex, indicating an improving trend with treatment (Figure 4a,i). GAA accumulation was significantly decreased in treated animals in all tissues tested and approaching heterozygous levels in the spinal cord and muscle (Figure 4b,d,f,h,j,l, Table S1). Note that some tissue samples were excluded due to poor tissue integrity, impacting protein extraction and/or accurate weighing.

**Figure 5:**
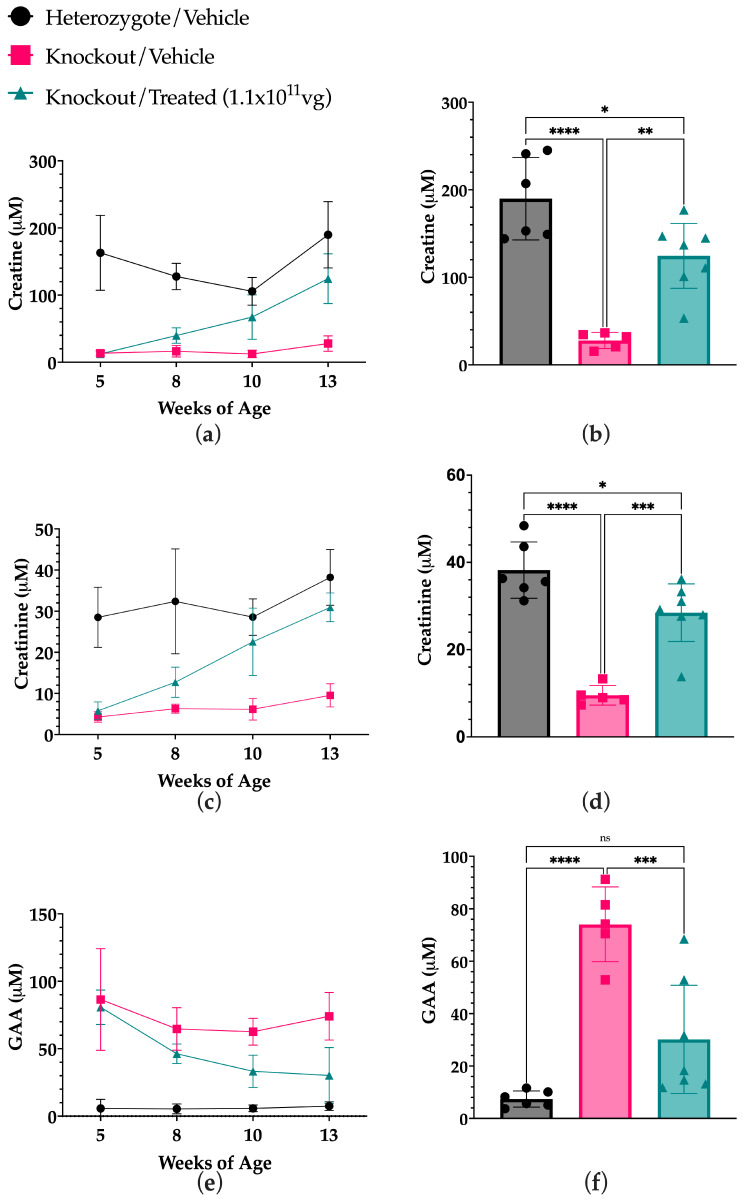
Cr and GAA content in serum collected from murine models of GAMT-D treated with scAAV9.hGAMT and vehicle controls determined by LC-MS/MS. Mice were treated with either 1 × 10^11^ vg per mouse of scAAV9.h*GAMT* or a vehicle solution via intrathecal lumbar puncture. Serum was collected at four timepoints at 5 weeks of age, 8 weeks, 10 weeks, and at endpoint at 13 weeks. (**a**). An increasing amount of Cr in the serum was observed over time from the 5-week baseline prior to treatment up to endpoint at 13 weeks of age. (**b**). A significant increase in Cr was observed in the serum of treated KOs compared to vehicle-treated KO controls at endpoint (**c**,**d**) The same trend for Cr was seen with creatinine levels. (**e**). A decreasing amount of GAA in the serum is observed over time from the 5-week baseline prior to treatment up to endpoint at 13 weeks of age. (**f**). A significant decrease in GAA was observed in the serum of treated knockouts compared to vehicle-treated knockout controls at endpoint (*n* = 5–7, * *p* < 0.05, ** p < 0.01, *** *p* < 0.001, **** *p* < 0.0001, ns = not significant). Data is represented as the mean (SD), a mixed effect analysis of a two-way ANOVA with Tukey’s and Dunnet’s multiple comparison test was ran (**a**,**c**,**e**), and a one-way ANOVA was used to compare groups at the endpoint (**b**,**d**,**f**).

There was a significant increase in Cr in the serum of treated mice over time from the baseline at 5 weeks, evident as soon as 8 weeks of age until the endpoint at 13 weeks (Figure 5a, Table 1), with the same trend seen for the downstream excreted product creatinine (Crn) (Figure 5c, Table 1). Similarly, a significant decreasing trend was observed for GAA levels in the serum, starting at 8 weeks of age up to the end point (Figure 5e, Table 1). Treated animals had significantly higher levels of serum Cr and Crn compared to vehicle-treated Kos, starting at 8 weeks of age, and this was stable until the endpoint, where there was no significant difference between the treated mice and heterozygous controls (Figure 5b,d, Table 2). GAA was also significantly lower than vehicle-treated knockout mice, observable at 10 weeks of age and at endpoint (Figure 5f, Table 2).

The Gamt KO models normally present with a lower body weight than their heterozygous or wildtype counterparts. Due to the short duration of the study, we were unable to distinguish any differences between the groups in body weight (Figure S1).

#### 2.2.2. Biodistribution of scAAV9.hGAMT Was Noted Throughout the Body and CNS After Intrathecal Administration with the Highest Copy Numbers Present in the Liver and Site of Injection in the Lumbar Spine

**Figure 6:**
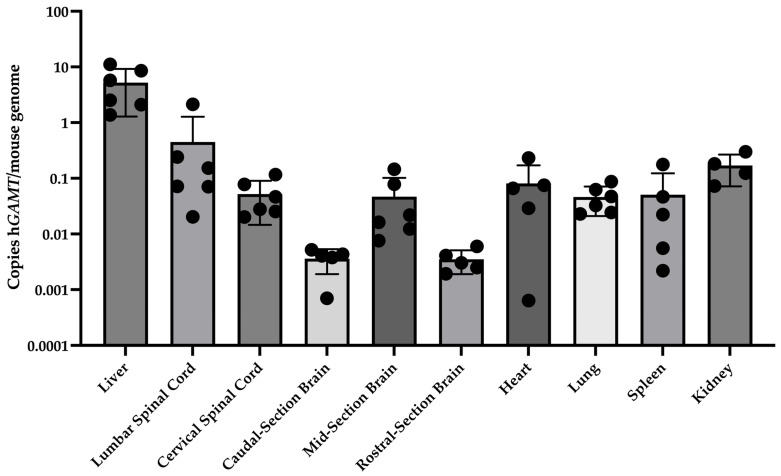
Biodistribution analysis shows that the highest levels of DNA copies of *hGAMT* are found in the liver, followed by the injection site in the spinal cord. Tissues were homogenized and subject to genomic DNA extraction for quantification via qPCR. Copy number was determined by comparing target copies to copies of the housekeeping gene Lamin B2 (*Lmnb2*). Data is represented by the mean (SD). The results of a one-way ANOVA comparing tissues showed that the liver had significantly higher copies than all other tissues tested (*p* < 0.0001) and that no other tissues showed significant differences.

Several tissues were collected, including three sections of the brain, two sections of spinal cord, and the peripheral organs—liver, heart, kidney, lung, and spleen—to evaluate biodistribution of the scAAV9.hGAMT vector via quantitative Polymerase Chain Reaction (qPCR). The housekeeping gene Lamin B2 (*Lmnb2)* was used to determine the approximate number of copies of *hGAMT* in treated mice per murine genome. The liver had significantly more *hGAMT* copies compared to all other tissues, consistent with what has been reported previously following intrathecal delivery of AAV9 vectors (Figure 6). Overall, the vector had a broad biodistribution and was found to be present in the CNS following the intrathecal injection.

#### 2.2.3. GAMT Protein Expression Is Restored in the Liver of Treated Animals of GAMT-D as Detected by Western Blot

**Figure 7:**
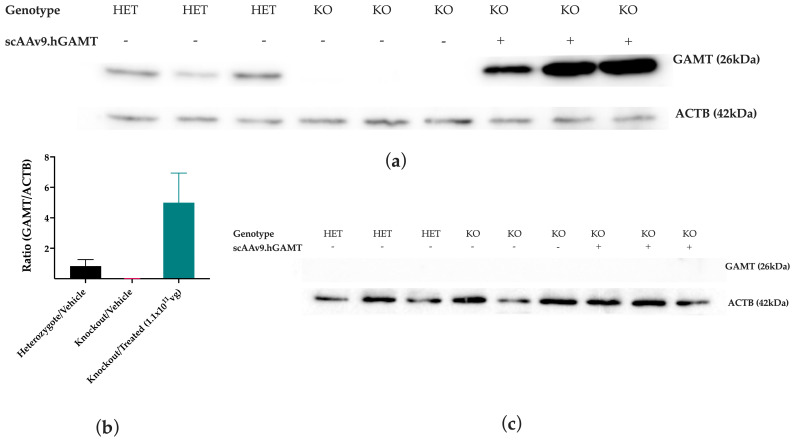
Western blot analysis of mid-section of the brain and liver tissues following treatment with *scAAV9.hGAMT*. Wells 1–3 are vehicle-treated heterozygote mice, wells 4–6 are vehicle-treated *Gamt* KO mice, and wells 7–9 are *Gamt* KO mice treated with 1 × 10^11^ vg of *scAAV9.hGAMT*. (**a**,**b**). Western blot carried out with protein isolated from the liver tissues loaded at 40 μg per well showed detectable hGAMT expression following treatment. The anti-GAMT antibody used is more specific to the human GAMT protein and therefore detects the murine GAMT at lower levels. Data is represented by the mean (SD) in panel B. (**c**). Western blot carried out with protein isolated from the mid-section of the brain loaded at 40 μg per well showed no detectable expression in any of the animals.

A Western blot was performed on protein lysates from tissue homogenates of the liver and the brain tissues to explore whether the vector treatment would result in detectable human GAMT expression. The antibody used for the Western blot is specific to human GAMT and, while cross-reactive with murine GAMT, has a lesser affinity for the murine protein. Since the intrathecal delivery of vectors in adult mice transduces <10% of brain cells, such as astrocytes and neurons [38], expectedly, there was no detectable expression of GAMT in the brain of treated mice or heterozygotes (Figure 7c). GAMT expression is typically low in the brains of both humans and mice, and given the antibody affinity, it was expected the heterozygous mice would have no detectable GAMT expression in the brain. scAAV9.hGAMT biodistribution in brain tissues and an analysis of intracellular Cr content showed Cr normalization even at lower copy numbers of h*GAMT* and low levels of protein expression in the brain, confirming therapeutic efficacy. However, the treated mice had detectable human GAMT expression in the liver (Figure 7a). This is indicative of the successful gene delivery of *GAMT* by the *scAAV9.hGAMT* vector, leading to protein expression in the liver, which is known to have a high level of uptake of AAV9 even following intrathecal administration.

## 3. Discussion

Cr plays a critical role in energy metabolism through the PCr/Cr cycle, which provides a buffer for ATP production that is particularly important in cells with high energy demands, such as skeletal muscle and neurons. GAMT-D is the most severe form of CDS due to the combined effect of creatine depletion and the accumulation of GAA, which has been found to impact brain cell development [39]. Although interventions with Cr supplementation, ornithine treatment, and arginine restriction have shown beneficial improvements, many symptomatic children continue to experience cognitive delays, impaired speech, and behavioural abnormalities [29,40]. The monogenic nature of the disease makes an ideal candidate for gene replacement therapy (GRT) and the recent addition of GAMT deficiency to newborn screening panels may allow for early intervention with GRT prior to symptom onset [30].

Here, we present the first gene therapy that is designed to target the CNS directly both in construct and in the route of administration to treat GAMT-D. A notable advantage to targeting the CNS directly with GRT is that the localized delivery allows for the dosage to be considerably lower than traditional systemic delivery. This effectively lowers the risk of hepatotoxicity and immune reactions, as well as the cost of the therapy for both the manufacturer and the patients. Based on a recent review, clinical dose selection for targeted delivery may range from as low as 5.8 × 10^9^ to a high dose of 7.5 × 10^15^ vg per patient, with the majority of clinical trials for targeted delivery using doses between 10^11^ and 10^13^ vg per patient [41]. Comparatively, systemic administration requires a 1000-fold increase from 10^14^ to 10^16^ vg per patient [41]. Our chosen dose of 1 × 10^11^ vg/mouse was based on assumptions regarding the translation of mouse CSF volume (approx. 0.035 mL) to humans (approx. 140 mL) [42]. We extrapolate that this equates to a 4000-fold difference between mice and humans for dosing; therefore, our 1 × 10^11^ vg dose in mice would equate to an approximate 4.5 × 10^14^ vg/adult patient, representing a below maximal dose (considered to be around 7.5 × 10^15^ vg for direct administration) [41]. In this short-term study, we observed significant improvements in intracellular Cr and GAA levels in several of the tissues and serum samples tested; however, not all tissues showed normalization to heterozygote levels. This indicates that it is warranted to test larger dosages in the future to determine the therapeutic window where maximal impact can be achieved without signs of toxicity.

It is well reported in the literature that AAV9 preferentially transduces the liver even following intrathecal administration, with better CNS biodistribution than systemic administration [42,43,44]. Our study results are consistent with these previous studies, with higher transduction of liver and an otherwise widespread distribution based on copy number [43,44]. In the target organ of the brain, we noted lower but widespread distribution copy numbers, as confirmed by qPCR; however, Western blot did not detect GAMT protein expression in any animal, consistent with other reports for intrathecal gene therapy delivery [42,45,46]. It is also worth noting that the 1.1 × 10^11^ vg/mouse is 2000-fold lower than that used in intravenous delivery for alternative vectors, highlighting the potential of localized delivery to achieve widespread results at low dosages. Including a ubiquitous promoter in the vector design leverages the ability of AAV9 to target both the liver and the CNS, albeit at a lower rate, with significantly lower intrathecal dosages achieving similar or improved biochemical benefits as compared to intravenous or liver-targeting therapies. GAMT-D is an ideal disease target for the transduction profile of the natural AAV9 serotype as endogenous *GAMT* is most highly expressed in the liver, and to a lesser extent in the brain [13,47]. Thus, by designing a scAAV9 vector to deliver the *GAMT* coding sequence, both the peripheral organs and brain, and the Cr production systems in the brain and periphery, can be restored via an intrathecal injection with low dosages that have improved safety profiles and are more accessible to patients.

It is unknown what level or percentage of GAMT enzyme activity is sufficient for the complete rescue of endogenous Cr production, and this may be answered by a future ‘dose-finding’ study. Heterozygotes in both humans and mice have a phenotypically normal presentation and therefore represent a good benchmark for rescue, hence why heterozygous mice were chosen as positive controls. RNA sequencing (scRNA-Seq) analysis of mouse tissue has shown that *Gamt* is expressed in several different types of brain cells, with the oligodendrocytes and cerebral cortical neurons showing the highest levels of expression in the mouse brain [13]. *Gatm* (encoding the AGAT enzyme) is highly co-expressed with *Gamt* in oligodendrocytes but not within neurons; overall, *Gatm* is expressed in the brain at higher levels than *Gamt.* The higher levels of *Gatm* expression may help explain why GAA may be significantly higher within the brain of GAMT-D patients and mouse models, as well as being elevated in many cases of Cr transporter disorder [48].

The lower levels of *GAMT* expression in the brain may allow for GRT to work more effectively, as high levels of transduction in all cell types are likely not required to achieve a therapeutic effect. GAA has been reported to be transported by SLC6A8 as well as other transporters in the SLC6A and SLC16A family, such as γ-Aminobutyric acid transporter 2 and the taurine transporter [10,11]. Therefore, the efficacy of the *scAAV9.hGAMT* vector will likely heavily rely on a cross-correction phenomenon within the brain, i.e., Cr made by one cell is taken up by the many surrounding cells [8,15]. A stronger therapeutic effect would likely be seen if cells with higher expression of SLC6A8 and other GAA transporters were transduced, such that accumulated GAA can more easily enter cells with a newly functional GAMT enzyme. Alternatively, if more cells expressing *GATM* (AGAT) are transduced, this may also more effectively reduce GAA accumulation. Additionally, while much less is understood about Cr export, the cross-correction within brain regions may also be dependent on the expression of this exporter to release Cr from GAMT-expressing cells for uptake by additional SLC6A8-expressing cells. Recently, the monocarboxylate transporter 12 (MCT12) has been suggested to be primarily responsible for Cr efflux in the liver and GAA efflux in the kidney [11,49,50]. The MCT12 transporter does not use active transport and instead uses passive transport against a concentration gradient to facilitate Cr and GAA efflux into the blood; however, this has not been well-characterized in the brain [11,49,50,51].

Taken together, this suggests that there could be significant biological variability following treatment with *scAAV9.hGAMT* based on the specific cellular transduction profile. This is not the case in the *in vitro* model study, as there is minimal variability in expression of AGAT and SLC6A8 in the HA1 cell population. Interestingly, an unexpected result was the complete reduction in intracellular GAA accumulation *in vitro* even with modest Cr increases, especially at the lower 1.25 µg plasmid dose. While it may be expected that if all available GAA substrate was consumed, Cr would, in turn, be significantly increased, creatine can be converted to both PCr (which was not measured) and Crn, which can be excreted into the extracellular space for waste removal. There may have been variability between cell populations in the rate of conversion of Cr to either PCr or Crn; however, it is not possible to be sure that this explains the lower Cr levels. Nonetheless, the results of the *in vitro* study determined that the designed construct was able to produce functional GAMT and, given that the complexity of an in vivo biological system would influence the availability of GAA and Cr, this was not deemed a critical concern.

GAA has been implicated to result in brain toxicity through several mechanisms, including increasing acetylcholine and oxidative stress, as well as increasing axonal hypersprouting [15]. Due to its similarity to GABA, these effects, and particularly the increased seizure activity, have been suggested to be due to the stimulation of GABA receptors by accumulated GAA [10,19]. It has also been shown that 10 µM long-term exposure to GAA (which is the approximate level found in the cerebral spinal fluid of GAMT-D patients) impaired developing rat brain cells in culture [15,39]. However, there is limited understanding of the level of GAA that may be toxic, and other studies have suggested that short-term GAA administration does not result in neurotoxic effects in vitro [52]. In relation to GRT for GAMT-D, it is unclear if a complete normalization in GAA would be required to achieve therapeutic efficacy or if a significant reduction would be sufficient to ameliorate the neurotoxic effects of GAA. It is also worth noting that these effects may be more potent in developing brains, and while diagnosis in utero is unlikely in most cases, this aligns with the suggestion that treatment as early as possible following birth would have the greatest impact on patients [27]. Both clinical data and recent understanding of the effects of Cr and related compounds on brain cells during development suggest there may be a critical period for GAMT-D treatment. *Gamt*-deficient mice were found to have reduced oligodendrocyte maturation and delayed myelination in the corpus collosum, and *Gamt* expression has been found to be highly regulated during development, with increased expression in the second post-natal week during active dendritogenesis and synaptogenesis [47,53]. Additionally, treatment in pre-symptomatic patients has achieved the best outcomes in reducing neurological symptoms in GAMT-D patients, leading physicians and advocates to push for GAMT-D to be added to newborn screening panels [27,29,30,54]. The identification of GAMT-D patients at birth would also allow for early intervention with gene replacement therapy, likely leading to more positive outcomes, as seen with earlier dietary treatments.

Early intervention may be ideal; however, many patients are not diagnosed until later in infancy and we therefore used 6-week-old adult mice in our study to determine if gene therapy treatment would have a significant impact on the biochemical phenotype when fully established. Our results determined that *scAAV9.hGAMT* is capable of restoring GAA levels in treated adult mice, often normalizing them to the levels of control mice. While the GAMT-D mouse model shows significant biochemical alterations in Cr and GAA, the phenotypic presentation is rather settled [20,55,56]. This is a limitation, given that human patients present with developmental delays, intellectual disabilities, seizures, and behavioural abnormalities. The severity of these phenotypes, however, is a spectrum that has not been effectively linked to genotype variants, likely due to the small number of reported patients and large number of private mutations [57]. Within the small diagnosed patient population, there are over 70 pathogenic variants of GAMT-D, with missense being the most common [7,57]. Other variant types include nonsense, deletion, truncating, splice-site errors, and frameshift mutations [7,25,29,57]. It has also been found that the severity of symptoms can be varied even across patients harbouring the same mutation, and since most pathogenic mutations result in no residual GAMT activity, it is difficult to draw any tangible conclusions regarding genotype/phenotype correlation [22]. The two most comment variants include the missense variant NM_000156.6(GAMT):c.59G>C (p.Trp20Ser), found predominantly in Portuguese patients, which results in a non-functional GAMT enzyme despite the detectable presence of GAMT protein by Western blotting [7,58]. Secondly, the synonymous variant NM_000156.6(GAMT):c.327G>A (p.Lys109=) contains two abnormal transcripts: one leading to the activation of a cryptic splice site in intron 2 and deletion of 146 bp, and another leading to a 44 bp insertion with altered splicing and skipping of exon 2. This has been identified in 30 unrelated GAMT-D patients [7,59].

The mouse model for GAMT-D was generated by Schmidt et al. in 2004 via the insertion of a neomycin cassette in exon 1 of the *Gamt* open reading frame (ORF) [55]. This insertion results in a premature stop codon, eliminating 210 of the 237 amino acids that make up the GAMT protein [55]. *Gamt* KO mice demonstrate a significant alteration in guanidino compound levels, similar to human patients, in which Cr and PCr are reduced in the brain, serum urine, and muscle, while GAA (or phosphorylated GAA) is significantly elevated [55,56,60,61,62]. Although the mouse model does not display certain behavioural phenotypes, GAMT-D, no matter the variant, is defined clinically by the presence of high GAA levels and, in most cases, lowered Cr and Crn. These biochemical alterations are thought to be the cause of the neurodevelopment symptoms; thus, treatment to control Cr and GAA levels is the most viable approach, regardless of the mutation variant. The neurological symptoms in patients may have a therapeutic window during development that, if missed, may not be fully reversible even if biochemical correction is achieved. It may be necessary to apply more sensitive behavioural measurements and cortical EEG recordings in the GAMT-D mouse models, which may have not been previously tested, in future studies with *scAAV9.hGAMT.* Additionally, investigating earlier treatment interventions, for example, in neonatal mice, may also be beneficial. 

## 4. Materials and Methods

### 4.1. Generation of GAMT Knockout in HAP1 Cells

Two guide gRNAs targeting either the noncoding region of GAMT exon 1 GAMTGRNA50nCDS-For 5′ CACCGGACCTCGATCGCGCGCCGCC and GAMTGRNA50nCDS-Rev 5′ AAACGGCGGCGCGCGATCGAGGTCC) or the coding region of exon1 (GRNA51CDS-For 5′-CACCGGTGCGTGTCCGCTGCGTCGT and GRNA51CDS-Rev 5′-AAACACGACGCAGCGGACACGCACC) were cloned into Cas9 and gRNA expression vector (PX458), as previously described [63]. Sequence-verified px458 gRNA50 and 51 expression plasmids were used to transfect one million HAP1 cells using GenJet Vers. II (Signagen, Frederick, MD, USA). Following 3-day expression, cells were treated with trypsin, diluted 10 cells/mL, and plated into 6 96-well plates. Cells (wells) no longer expressing GAMT were identified by in-cell Western blotting using a polyclonal antibody against GAMT [64]. For staining, media from cells were removed and cells were washed twice with PBS, then fixed with 3.8% paraformaldehyde (Electron Microscopy Services, Hatfield, PA, USA) in PBS for 20 min at room temperature. Cells were washed with PBS, then permeabilized with PBS containing 0.1% TritonX 100, followed by blocking with TBST (Tris Balanced Saline containing TBST, 50 mM Tris pH 7.5 150 mM NaCl, 0.1% Tween 20) containing 5% non-fat dry milk powder for 2 hrs. Blocking buffer was removed, and cells were incubated with 25 μL of GAMT Ab (1/1000) diluted in TBST 5% milk powder overnight at 4 °C. The next day, cells were washed with TBST four times, at 200 μL, followed by the addition of 1/10000 dilution Goat Anti-Rabbit IgG conjugated to horseradish peroxidase (Cell Signalling Technologies, Danvers, MA, USA). Following incubation for 1 hr at 4 °C, cells were washed with four changes in TBST. GAMT Ab binding was visualized using the luminescent substrates (Biorad, Hercules, CA, USA) and imaged using the high-sensitivity Chemiluminescent channel on the Biorad ChemiDoc MP imaging system (Biorad, Hercules, CA, USA). Wells that showed no luminescence, i.e., no binding of antiGAMT Ab, represented potential GAMT knockout clones. These wells were checked under the microscope to ensure that they contained cells and, following verification, were expanded. Putative GAMT knockout clones were further confirmed by Western blotting; those with no detectable GAMT protein were selected for final confirmation by sequencing. Several clones were selected for the sequencing of PCR product encompassing part of the GAMT promoter and exon 1 using primers (GAMTEx1For1: 5′-GTGCTGACAGATGAGGAATCTATG and GAMTEx1Rev1: 5′-CGCGTGCATATAGGGGGTCT). Clone 5B6 was further subcloned by limiting cell dilution to isolate and expanding colonies grown from single cells.

### 4.2. Cell Culture

Cells were maintained in Iscove Modified Dulbecco Medium (IMDM) substituted with 1% penicillin–streptomycin (Wisent, Saint-Jean-Baptiste, QC, Canada) and 10% dialyzed fetal bovine serum (FBS) (Wisent, Canada). FBS was placed in a dialysis membrane (12–14 Kda cutoff) (Fisher Scientific, Ottawa, ON, Canada) and dialysed against 0.9% saline solution to remove small molecules such as Cr, as previously described [65]. Cells were grown in the media for at least two weeks prior to transfection to ensure cells were free of media-related Cr. Cells were maintained in an incubator at 37 °C and 5% CO_2_.

### 4.3. Plasmids and Vectors

The *GAMT* vector includes the codon optimized human *GAMT* cDNA sequence. This construct is under the control of the synthetic JET promoter and followed by a poly-adenylation signal (Figure S2). The entire sequence is flanked by inverted terminal repeats (ITR) to allow for packaging into the self-complementary AAV9 vector, with the 3′ ITR having a mutated terminal resolution site to allow for self-complementary folding [66]. The designed vector was synthesized as a plasmid with codon-optimized transgene sequences for optimal expression (Biobasics, Markham, ON, Canada). The plasmid was transformed by addition to competent *E. coli* bacterial cells and isolated by miniprep in accordance with the kit protocol (QIAprep Spin Miniprep Kit, Qiagen, Hilden, Germany) for use in transfection. DNA concentration and purity were determined using a Nanodrop 2000 (Thermo Fisher Scientific, Waltham, MA, USA). Plasmids were sent to Aldevron, LCC (Fargo, ND, USA) for larger plasmid prep and to UNC Vector Core for viral vector preparation (UNC Vector Core, Chapel Hill, NC, USA).

### 4.4. Transfections

For transfection, cells were seeded in a 6-well plate at approximately 500,000 cells per well determined by manual cell counting using Trypan Blue (Gibco, Thermo Fisher Scientific, Waltham, MA, USA). The *GAMT* plasmids were introduced to the cells by Lipofectamine 3000 (Invitrogen, Thermo Fisher Scientific, Waltham, MA, USA)-mediated transfection according to the manufacturer’s protocol at a low dose of 1.25 μg and a high dose of 3.75 μg. Cells were treated with plasmids containing the human *GAMT* or murine *Gamt* construct or a CAG-GFP plasmid to provide a visualization of transfection efficiency. After 48 h, cells were subject to protein or RNA isolation for their respective analyses.

### 4.5. Animal Models and In Vivo Study Design

The GAMT-D mice (B6J.129-Gamttm1Isb) were generated at the University Medical Centre Hamburg-Effendorf and were provided by Dr. Andreas Schulze [55]. Animals were maintained on a 12 hr light cycle from 7 a.m. to 7 p.m. Mice were bred in-house and ear-notched for identification. All experimental protocols and procedures were performed in accordance with the Canadian Council on Animal Care and were approved by the Queen’s University Animal Care Committee. Animals were provided with bedding and plastic igloos for enrichment. For the in vivo proof of concept study, a total of 18 single animals were injected intrathecally with 1.1 × 10^11^ vector genomes (vg) per mouse of the *scAAV9.hGAMT* vector or a vehicle control at 6 weeks of age (Table 3). Vectors were prepared in 1X PBS with 5% sorbitol for the appropriate dosage while vehicle injections were performed with 1X PBS with 5% sorbitol only (UNC Vector Core, Chapel Hill, North Carolina). A total number of five single animals per cohort was targeted due to previous published biochemical findings between heterozygous and knockout animals. An a priori test was carried out on G*Power based on this previously published data [55,67]. Two additional animals were included in the treatment group to control for potential injection uncertainty. Six animals were included in the heterozygous control group due to genotype availability. Following genotyping, animals were randomly placed in treatment groups with all heterozygous animals receiving vehicle injections and knockout animals assigned vehicle or treatment. Animals were divided equally between male and female mice when possible. Researchers were not blinded to the study group allocation. Animals were singly housed and moved only when required for treatments, blood collections, and standard cage changes to minimize confounders. Animals received treatment in the same order and were all subject to daily handling for oral gavage.

### 4.6. Genotyping

Genotyping was performed by standard PCR on DNA extracted from ear notches using the Extracta DNA prep for PCR kit (VWR, Radnor, PA, USA). Primers were as follows: forward primer 1: 5′-GGTCTCCCAACGCTCCATCACT-3′, reverse primer: 5′-CCTCAGGCTCCCACCCACTTG-3′ and forward primer 2: 5′-AGGCCTACCCGCTTCCATTG-3′. Samples were prepared using the Advanced 2X HS-Red Taq PCR kit (Wisent, Canada).

### 4.7. Intrathecal (IT) Injections

The protocol for performing intrathecal injections was adapted from the previous literature [44,68]. At 6 weeks of age, mice were anesthetized by inhalation of isoflurane at 4–5% for induction and maintained at 1–3% for injection. The mice were placed with their head in a nose cone with their hips elevated by a 15 mL conical tube (S1). The back of the mouse was shaved and sterilized and the location between L5 and L6 was palpated to mark the injection spot. A Hamilton syringe (Hamilton, Reno, NV, USA) with a 30-gauge needle (Hamilton, Reno, NV, USA) was loaded with the vector at a volume of 10 μL for a dose of 1.1 × 10^11^ vector genomes (vg) per mouse. The syringe was inserted at a 90° angle from the spine and proper penetration was indicated by a tail flick—a movement of the tail in the shape of an S or tail vibrations during injection. The mice were recovered in a clean cage and monitored for several minutes after injections. Anesthetization was used to reduce suffering and distress and to lower the incidence of injection-related injury or paralysis.

### 4.8. Immunosuppression

To reduce the immune response against the vector, mice received an immunosuppression regimen from 5 weeks of age to 13 weeks of age. Rapamycin (LC-Laboratories, Woburn, MA, USA) at a loading dose of 3 mg/kg, followed by 1 mg/kg/day and Prednisone (Sigma Aldrich, St. Louis, MO, USA) at 0.24 mg/kg/day, given daily via oral gavage for the duration of this study. To minimize potential confounders, animals were all gavaged at the same time in the morning each day.

### 4.9. Blood Collections

Blood collections were performed monthly by collecting approximately 100 μL of blood from the saphenous vein using capillary tubes (Kent Scientific, Torrington, CT, USA). The serum was collected by separation from the blood sample through centrifugation.

### 4.10. Euthanization

Tissue samples were collected at the designated short-term endpoint of 13 weeks; a humane endpoint was not included in the study as this strain of mice lives a normal life-span. The mice were euthanized by CO_2_ asphyxiation, after which a cardiac puncture was performed. Mice were then perfused with 10 mL of 1X PBS and tissues were harvested.

### 4.11. Quantitative Polymerase Chain Reaction (qPCR)

Copy numbers of the *scAAV9hGAMT* vectors and mouse genomic DNA were determined by qPCR, as previously described [42]. Total DNA was extracted from tissues using the Geneaid™ gSYNC DNA Extraction Kit (Frogga Bio Inc., Concord, ON, Canada) and total DNA concentration was determined using a Nanodrop 2000 (Thermo Fisher Scientific, Waltham, MA, USA). Quantitative PCR (qPCR) reactions were carried out using the SsoAdvanced Universal SYBR Green Supermix (BioRad, Hercules, CA, USA) on a Biorad CFX96 Touch Real-Time PCR Detection System. Plasmid DNA (*hGAMT)* was used as the standard for quantitation of the vector with the following primers: F: GGATTCTTGGGAAGCCCGTG and R: GGGGTGATCATCTGGGGGAA. Mouse genomic DNA from wildtype liver tissue was purified as a standard for mouse genomic DNA quantitation using the Lamin B2 (*Lmnb2*) gene with the following primers: F: GGACCCAAGGACTACCTCAAGGG and R: AGGGCACCTCCATCTCGGAAAC. To determine the relative copy number variation in the viral vectors in each organ, the values were reported as double-stranded copies of the GAMT vector per double-stranded copies of the mouse LaminB2 locus. This will provide an approximate measure of the vector genome copies per diploid mouse genome found in the assessed tissues. Gene expression was analyzed by isolation of RNA for use in RT-qPCR. RNA isolation from cells was carried out with the GeneJET RNA Purification kit (Thermo Fisher Scientific, Waltham, MA, USA) cDNA was synthesized using the Quantitect Reverse Transcription kit (Qiagen, Germany). The forward and reverse primers for *hGAMT* were used as described above. RNA18S was used as the reference gene for the in vitro analysis to quantify gene expression with the following primers: F: GTGGAGCGATTTGTCTGGTT and R: AACGCCACTTGTCCCTCTAA. The Delta–Delta Cq method was used to quantify gene expression. All MIQE guidelines were followed to ensure best qPCR practices [69].

### 4.12. Liquid Chromatography Tandem Mass Spectrometry (LC-MS/MS)

The instrumentation and parameters for guanidino compounds’ detection and quantification were used as previously described [70]. Sample extraction for guanidino compounds from tissues was as follows. Liquid nitrogen frozen tissue weighing 10–150 mg were homogenized in 250–1000 mL of water based on weight on the Omni Bead Ruptor Elite (Omni, Kennesaw, GA, USA) using 2.8 mm ceramic beads at a speed of 2.85 m/s for two cycles of 20 s, with 10 s in between cycles. Samples were then sonicated at 20% power for 10 s, twice. To precipitate proteins, tissue homogenates were treated with TCA at a final concentration of 7.5%, vortexed, and spun down at 13,000× rpm for 5 min. HAP1 cells were processed as previously described [70]. Derivatization was initiated by mixing either 10 µL of serum, 10 µL of supernatant from cleared tissue homogenates, or 100 µL of cell lysate, with 10 µL of the internal standard and 500 µL methanol. Samples were then vortexed and spun down at 13,000× rpm for 5 min. The supernatants were transferred into glass test tubes and loaded onto the Microvap (Organomation, Berlin, MA, USA) to evaporate the excess of solvent at 37 °C. The dry residues were dissolved in 100 µL buthanol·HCL (3 M) by vortexing and incubated at 60 °C for 30 min. After cooling to room temperature, derivatized samples were transferred onto the Microvap to evaporate the excess of solvent at 37 °C. Finaly, dry residues were resuspended in 700 µL methanol and transferred into a 2 mL glass vial for the injection on LC-MS/MS. The raw data reads in μM were normalized to the protein concentration in μmol/μg for cells and for tissue weight in nmol/g.

### 4.13. Western Blotting

To extract protein lysate from the cellular models, 400 μL of 1X radioimmunoprecipitation assay (RIPA) buffer (Cell Signalling Technology, Danvers, MA, USA) was added to each well and briefly incubated on ice. The cells were scraped into 1.5 mL Eppendorf tubes and sonicated (20% power, 10 s/sample, twice). Cell debris was removed by centrifugation. Tissue samples were weighed out and added to a bead tube for homogenization with a RIPA buffer at a ratio of 1 mL:100 mg of tissue. Samples were processed on the Omni Bead Ruptor Elite (Omni, Kennesaw, GA, USA) using 2.8 mm ceramic beads at a speed of 4.00 m/s for two 20 s cycles separated by 10 s. The homogenate was incubated in the RIPA buffer for 10 min on ice. The tissues were then sonicated (20% power, 10 s/sample, twice) and debris was removed by centrifugation at max speed before collection of the supernatant. Protein concentration was determined using the Pierce BCA Protein Assay Kit following the manufacturer protocol (Thermo Fisher Scientific, Waltham, MA, USA).

Western blots were performed in accordance with most standard protocols. Briefly, 30 μg (cell lysate) or 40 μg (tissue lysate) of protein was loaded into each well, with one well containing a ladder (Precision Plus Protein™ Kaleidoscope™ Prestained Protein Standards, BioRad, Hercules, CA, USA), and ran on a 12.5% polyacrylamide gel (SDS-PAGE). The proteins were transferred to a nitrocellulose membrane, followed by blocking with a 5% skim milk solution, and then incubated overnight with the primary anti-GAMT rabbit polyclonal antibody (Thermo Fisher, Bethyl Laboratories, Waltham, MA, USA). Following several washes with 1XTBST, the secondary antibody (Goat anti-rabbit IgG HRP, Invitrogen, Thermo Fisher Scientific, Waltham, MA, USA) was added, followed by another set of washes. Proteins were visualized by the chemiluminescent detection method using Immobilon Western chemiluminescent HRP substrate reagents (Millipore Sigma, Burlington, MA, USA). The Western blot was imaged using the Azure Biosystems C600 imaging system. The β-Actin protein was used as an internal control to show equal protein loading between wells. The membrane was washed following imaging for the GAMT target protein, and then incubated with the primary anti-actin antibody produced in rabbit (Sigma Aldrich, St. Louis, MO, USA) overnight and imaged following the same steps as above for secondary antibody staining. ImageJ software (https://imagej.net/ij/; accessed 16 April 2022) was used to quantify bands as a ratio of GAMT target to the ACTB internal control.

### 4.14. Statistical Analysis

All statistical analyses were performed in Graph Pad Prism 9. For all Cr and GAA intracellularly and in tissues, an ordinary one-way ANOVA with Tukey’s multiple comparison test was used to compare treatment groups. In the case of serum, a repeated measures mixed effects analysis was ran with Tukey’s multiple comparison test to compare cohorts at each timepoint and a Dunnet’s multiple comparison test to compare baseline levels to other timepoints within cohorts. Fold-change was determined by subtracting the 5-week baseline from matched data points at each timepoint and then computing the ratio of the change over the baseline value. Data was determined to be normally distributed by the Shapiro–Wilk normality assessment; the cohort size was too small for alternative normality tests to be performed.

## 5. Conclusions

Overall, this study has shown, for the first time, the potential for a curative gene therapy approach to treat GAMT-D using a self-complementary AAV9 vector to deliver the human *GAMT* gene via intrathecal delivery. Direct CNS administration allows for the use of lower doses and a more targeted therapeutic effect, resulting in significant biochemical improvements in guanidino compound levels in the brain and periphery. This study lays the foundation for further investigation in pre-clinical studies to determine the most appropriate dose range and evaluate durability in a longer-term study in GAMT-D murine models. Additional studies to investigate alternative routes of administration, including intravenous delivery, as well as ages of administration, may further add information applicable to the clinical translation potential of the vector. Exploration of the immune tolerance and toxicology profile of *scAAV9.hGAMT* are also ongoing to create a basis for clinical translation and investigation in human patients in the future. Given the recent increase in both newborn screening and emerging investigative gene therapies for creatine deficiency, efforts including caregivers and healthcare professionals have been made to facilitate the ideal investigational outcomes for clinical trials for evaluating both new and existing therapeutic strategies [71]. The potential of gene therapy for GAMT-D is significant as it may represent a more curative pharmacological approach to treating a disease with minimal therapeutic options. The addition of GAMT to several newborn screening panels also provides an early treatment window for a future approved gene therapy to have the most significant therapeutic benefit. This proof-of-concept study is a critical foundation for the designed scAA9.hGAMT with the aim of generating a package of data that can ultimately be translated to clinical development.

## Figures and Tables

**Table 1:** Statistical significance summary of Cr, GAA, and creatinine concentrations in the serum from baseline to endpoint. This table corresponds to the results in Figure 5. Statistical analysis was performed by applying a repeated measures mixed effects model of a two-way ANOVA with a Dunnett’s multiple comparison test, using 5 weeks as the control comparison group.

	Creatine								
Time Comparison		Het/Vehicle		KO/Vehicle			KO/Treated (1.1 × 10^11^ vg)		
	Summary	*p*-value	Fold-Change	Summary	*p*-value	Fold-Change	Summary	*p*-value	Fold-Change
5 vs. 8	ns ^a^	0.3324	−0.1821	Ns	0.5544	0.2648	**	0.0031	2.118
5 vs. 10	ns	0.0992	−0.3465	Ns	0.8523	0.0612	*	0.0289	3.995
5 vs. 13	ns	0.4497	0.0686	Ns	0.1735	1.286	**	0.0014	8.817
	GAA								
	Het/Vehicle			KO/Vehicle			KO/Treated (1.1 × 10^11^ vg)		
	Summary	*p*-value	Fold-Change	Summary	*p*-value	Fold-Change	Summary	*p*-value	Fold-Change
5 vs. 8	ns	0.9695	0.2039	Ns	0.1652	−0.1739	**	0.0026	−0.4095
5 vs. 10	ns	0.9993	0.0555	Ns	0.1003	−0.2105	**	0.0017	−0.5750
5 vs. 13	*	0.0474	0.4610	Ns	0.3175	−0.1953	**	0.0059	−0.6163
	Creatinine								
	Het/Vehicle			KO/Vehicle			KO/Treated (1.1 × 10^11^ vg)		
	Summary	*p*-value	Fold-Change	Summary	*p*-value	Fold-Change	Summary	*p*-value	Fold-Change
5 vs. 8	ns	0.8922	0.1213	Ns	0.1818	0.4691	*	0.0201	1.426
5 vs. 10	ns	>0.9999	0.0287	Ns	0.3289	0.2941	*	0.0121	3.338
5 vs. 13	ns	0.1995	0.4314	Ns	0.1846	1.401	***	0.0001	5.042

^a^—ns (not significant).; * *p* < 0.05; ** *p* < 0.01; *** *p* < 0.001.

**Table 2:** Statistical significance summary of serum Cr, GAA, and Crn at each timepoint compared between each group. This table corresponds to the results in Figure 5. Statistical analysis was performed by applying a repeated measures mixed effects model of a two-way ANOVA with Tukey’s multiple comparison test. All groups were compared with all other groups at each timepoint.

Creatine								
	5 Weeks		8 Weeks		10 Weeks		13 Weeks	
Het/Vehicle vs. KO/Vehicle	**	0.0069	****	<0.0001	***	0.0001	***	0.0007
Het/Vehicle vs. KO/Treated	**	0.0066	****	<0.0001	ns	0.0847	ns	0.0563
KO/Vehicle vs. KO/Treated	ns ^a^	0.79	**	0.0053	*	0.015	**	0.0013
GAA								
	5 Weeks		8 Weeks		10 Weeks		13 Weeks	
Het/Vehicle vs. KO/Vehicle	*	0.018	***	0.0007	****	<0.0001	***	0.0008
Het/Vehicle vs. KO/Treated	****	<0.0001	****	<0.0001	**	0.0028	ns	0.0799
KO/Vehicle vs. KO/Treated	ns	0.8441	ns	0.0617	**	0.0018	**	0.0051
Creatinine								
	5 Weeks		8 Weeks		10 Weeks		13 Weeks	
Het/Vehicle vs. KO/Vehicle	**	0.0074	*	0.0101	****	<0.0001	****	<0.0001
Het/Vehicle vs. KO/Treated	**	0.0025	*	0.0235	ns	0.2788	ns	0.0933
KO/Vehicle vs. KO/Treated	ns	0.2791	*	0.0126	**	0.0066	****	<0.0001

^a^—ns (not significant); * *p* < 0.05; ** *p* < 0.01; *** *p* < 0.001; **** *p* < 0.0001.

**Table 3:** In vivo study design.

Cohort	Genotype	Treatment	Number of Animals (n)
1	*Gamt* +/−	Vehicle	6
2	*Gamt* −/−	Vehicle	5
3	*Gamt* −/−	1.1 × 10^11^ vg *scAAV9.hGAMT*	7

## Data Availability

All data necessary are provided with this report. Raw data used for statistical analysis are available upon reasonable request from Dr. J.S. Walia.

## Supplementary Materials

The following supporting information can be downloaded at: https://www.mdpi.com/article/10.3390/ijms27021035/s1.

## Abbreviations

The following abbreviations are used in this manuscript:

AAV	adeno-associated virus
AAV9	adeno-associated virus serotype 9
ACTB	beta actin
ADP	adenosine diphosphate
AGAT/*GATM*	L-arginine:glycine amidinotransferase
ANOVA	analysis of variance
ATP	adenosine triphosphate
BBB	blood–brain barrier
CCDS	cerebral creatine deficiency syndromes
CNS	central nervous system
Cr	creatine
Crn	creatinine
DNA	deoxyribonucleic acid
FBS	fetal bovine serum
GAA	guanidinoacetate
GABA	gamma-aminobutyric acid
GAMT	Guanidinoacetate methyltransferase
GAMT-D	Guanidinoacetate methyltransferase deficiency
GFP	green fluorescent protein
GRT	gene replacement therapy
HET	heterozygote
ITR	inverted terminal repeat
kDa	kilodalton
KO	knockout
LC-MS/MS	liquid chromatography tandem mass spectrometry
MCT12	monocarboxylate transporter 12
qPCR	quantitative polymerase chain reaction
RNA	ribonucleic acid
RT-qPCR	reverse transcription quantitative polymerase chain reaction
SD	standard deviation
SLC6A8/CTR	creatine transporter
vg	vector genomes
WT	wildtype
