# Supplementary material for: A CNS-Directed, AAV9 Gene Therapy Restores Expression and Biochemical Function of Guanidinoacetate Methyltransferase in Models of GAMT Deficiency
Source: Int J Mol Sci. 2026 Jan 20;27(2):1035. doi: 10.3390/ijms27021035 (PMC12842229; doi:10.3390/ijms27021035)
Supplement: Supplementary file 1 [file ijms-27-01035-s001.zip › ijms-4088053-supplementary.pdf]

Table S1. Significance summary of tissue Cr and GAA between all cohorts. This table corresponds to the results in figure 4. Statistical analysis was done by applying and ordinary one-way ANOVA with a Tukey's multiple comparison test for each organ. All groups were compared with all other groups.

	Cerebral Cortex		Cerebellum		Olfactory Bulb		Lumbar Spinal Cord	
**Creatine**								
Het/Vehicle vs. KO/Vehicle	*	0.023	***	0.0002	*	0.0129	****	<0.0001
Het/Vehicle vs. KO/Treated	ns	0.4827	ns	0.1263	*	0.0437	****	<0.0001
KO/Vehicle vs. KO/Treated	ns	0.1203	**	0.0064	ns	0.6996	*	0.014
**GAA**								
Het/Vehicle vs. KO/Vehicle	****	<0.0001	***	0.0003	*	0.0312	****	<0.0001
Het/Vehicle vs. KO/Treated	ns	0.2234	*	0.0256	ns	0.7269	ns	0.4005
KO/Vehicle vs. KO/Treated	***	0.0003	*	0.0495	ns	0.1423	****	<0.0001

Cervical Spinal Cord		Muscle		Liver	
***	0.0007	*	0.0161	****	<0.0001
**	0.0068	ns	0.7001	ns	0.0553
ns	0.3143	ns	0.0574	***	0.0007
****	<0.0001	****	<0.0001	****	<0.0001
ns	0.0813	ns	0.9868	*	0.017
****	<0.0001	****	<0.0001	***	0.0009

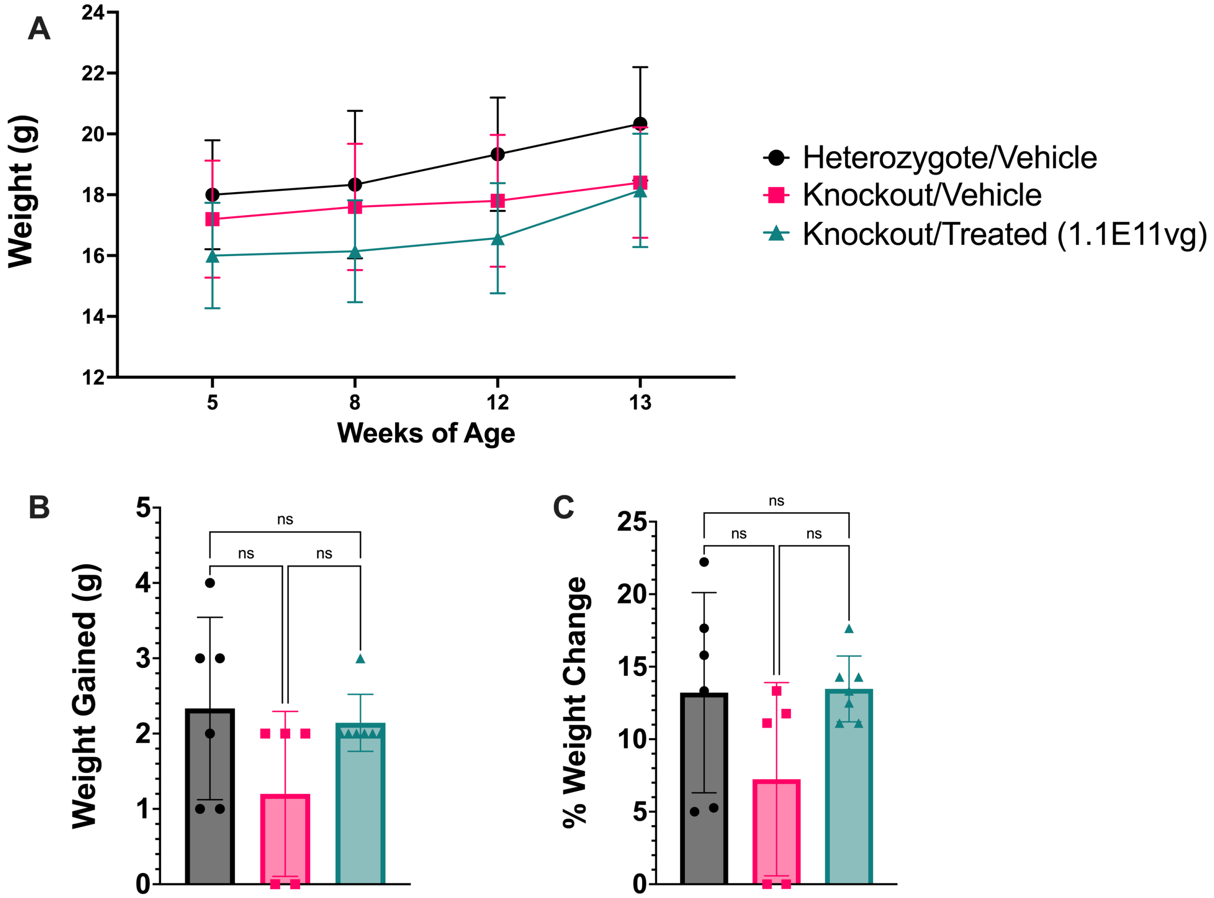

Figure S1. No differences in weight at any point were obtained between any of the groups. A. Weight measurements in grams were taken at a baseline of 5 weeks of age, then 8, 12 and the endpoint of 13 weeks of age. No statistically significant differences were observed between any of the groups (n=5-7, $p > 0.05$). **B.** Heterozygotes and treated mice gained an average of 2.3 and 2.1g respectively over the study duration while knockout mice gained an average of 1.2 grams. **C.** Heterozygotes and treated mice gained an average of 13% body weight over the study duration while untreated knockout mice gained an average of only 7%.

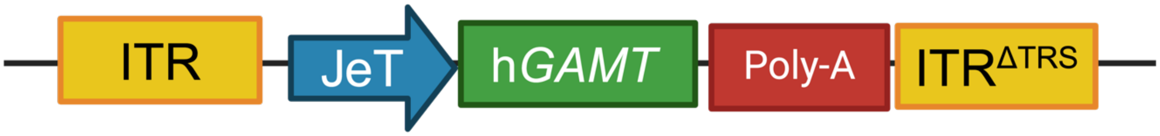

Figure S2. Construct map of scAAV9.hGAMT. The codon optimized human GAMT coding sequence is controlled by the synthetic ubiquitous JeT promoter and followed by a poly-adenylation signal flanked between two inverted terminal repeats for packaging into the scAAV9. The downstream ITR has a mutated terminal resolution site (TRS) to allow for self-complimentary formation.
